# Six-Month Outcomes of Endoscopic Intragastric Balloon Therapy: A Prospective Single-Center Study

**DOI:** 10.5152/tjg.2025.25043

**Published:** 2025-05-20

**Authors:** Mahmut Yüksel, Kerem Kenarlı, Fırathan Sarıaltın, Emine Sena Sözen, Ahmet Akbay, Erdoğan Deniz, Kübra Köken, Çağdaş Erdoğan, Mevlüt Hamamcı, Hasan Tankut Köseoğlu

**Affiliations:** 1 Department of Gastroenterology, University of Health Sciences, Ankara City Hospital, Ankara, Türkiye; 2 Department of Radiology, University of Health Sciences, Ankara City Hospital, Ankara, Türkiye; 3 Department of Internal Medicine, University of Health Sciences, Ankara City Hospital, Ankara, Türkiye

**Keywords:** Obesity, intragastric balloon, endoscopic therapy, weight loss, metabolic outcomes

## Abstract

**Background/Aims:**

Obesity is a growing global health challenge associated with increased morbidity and mortality. Intragastric balloon therapy has emerged as a minimally invasive alternative for weight management in patients unsuitable for bariatric surgery or as a bridging intervention. This study evaluates the efficacy, safety, and metabolic impacts of intragastric balloon therapy in a prospective cohort.

**Materials and Methods:**

A total of 65 patients underwent endoscopic intragastric balloon therapy between October 2023 and January 2025. Comprehensive baseline evaluations included body weight, body mass index, and computed tomography analysis of visceral and subcutaneous fat tissue thickness. Patients were followed up at 2 and 6 months post procedure. Statistical analysis was performed to assess weight loss, metabolic changes, and safety outcomes.

**Results:**

Of the initial cohort, 62 patients (mean age: 37.89 years; 75.4% female) completed the study. The median body weight decreased significantly from 104.5 kg pre-procedure to 88 kg at 6 months (*P* < .001). Body mass index values similarly declined, with a mean reduction from 37.59 to 32.79 kg/m^2^ (*P* < .001). Significant decreases in subcutaneous and visceral fat tissue thickness were observed (*P* < .001 and *P* = .032, respectively). Only 1 patient (1.5%) required early balloon removal due to nausea, with no other major complications reported.

**Conclusion:**

Intragastric balloon therapy is an effective and safe intervention for short-term weight loss, with significant reductions in adipose tissue. Further research is needed to evaluate its long-term efficacy and metabolic benefits.

**Main Points**

- This study demonstrates that intra-gastric balloon therapy is an effective short-term intervention for obesity management, achieving significant reductions in body weight over 6 months. The therapy also reduces subcutaneous and visceral fat tissue thickness, contributing to improved metabolic health.
- Intra-gastric balloon therapy was found to be safe, with no major complications reported apart from one case of severe nausea requiring early balloon removal.
  The study highlights the importance of patient education and monitoring to mitigate side effects and improve tolerability.
- As a minimally invasive and reversible option, IGB therapy provides an alternative for patients ineligible for bariatric surgery or requiring preoperative weight reduction.

## Introduction

The global increase in the prevalence of overweight and obesity has become a critical public health challenge, largely driven by urbanization and sedentary lifestyles.^1^ By 2030, it is projected that the number of adults affected by obesity will surpass 1 billion, doubling the prevalence recorded in 2010.^2^ Obesity is strongly associated with increased all-cause mortality, primarily due to its links with cardiovascular diseases and cancer.^3^ As such, the development and expansion of effective treatment options for obesity are imperative.

Obesity management traditionally relies on lifestyle modification, pharmacotherapy with anti-obesity medications, and bariatric or metabolic surgery. Lifestyle modification, including caloric restriction, increased physical activity, and structured behavioral interventions, remains the cornerstone of first-line treatment.^4^ However, even high-intensity interventions typically achieve only modest and often unsustainable weight loss, with few individuals maintaining a reduction of ≥5% of body weight in the long term.^5^

Recently, glucagon-like peptide-1 receptor agonists (GLP-1RAs) have emerged as a promising pharmacological therapy, demonstrating superior weight loss compared to earlier anti-obesity medications.^6^ Despite their potential, the widespread adoption of GLP-1RAs is hindered by factors such as high costs, supply limitations, insurance coverage issues, and tolerability concerns.^7^ Moreover, uncertainties regarding their long-term safety and efficacy persist, particularly the risk of irreversible gastrointestinal motility disorders.^8^ Consequently, bariatric and metabolic surgery remain the most effective interventions for achieving substantial and sustained weight loss, offering improvements in quality of life.^9^^,^^10^

For patients in whom surgical interventions are unsuitable due to medical risks or apprehension about surgery, endoscopic and medical management options are increasingly prioritized. Emerging endoscopic approaches for obesity management include space-occupying devices, restrictive techniques, bypass liners, aspirational therapy, and other innovative modalities.^11^ Among these, intra-gastric balloons (IGBs) represent a well-established space-occupying device, initially introduced in 1982 based on observations of bezoar-induced satiety. Intra-gastric balloons have demonstrated efficacy as a short-term adjunct and bridge to comprehensive obesity management.^12^ However, with most studies reporting follow-up periods of less than 12 months, the long-term impact of IGBs on weight loss and obesity-related comorbidities remains uncertain.^13^

In light of these considerations, various innovative balloon models and procedures have been developed, offering alternative strategies for obesity management. To contribute to the growing body of evidence, this study aims to evaluate the outcomes of patients who underwent IGB therapy at the institution, providing insights into its efficacy and role in clinical practice.

## Materials and Methods

### Study Population

We included patients of all genders, aged over 18 years, with obesity (body mass index [BMI] ≥ 30) and a suitable indication for IGB therapy between October 2023 and January 2025. Pre-procedure clinical gastroenterological and dietetic evaluations were performed by experts. Key parameters, including glycated hemoglobin (HbA1c), total cholesterol, low-density lipoprotein (LDL), body weight, BMI, and Homeostatic Model Assessment of Insulin Resistance (HOMA-IR) levels, were recorded.

**Figure 1.**
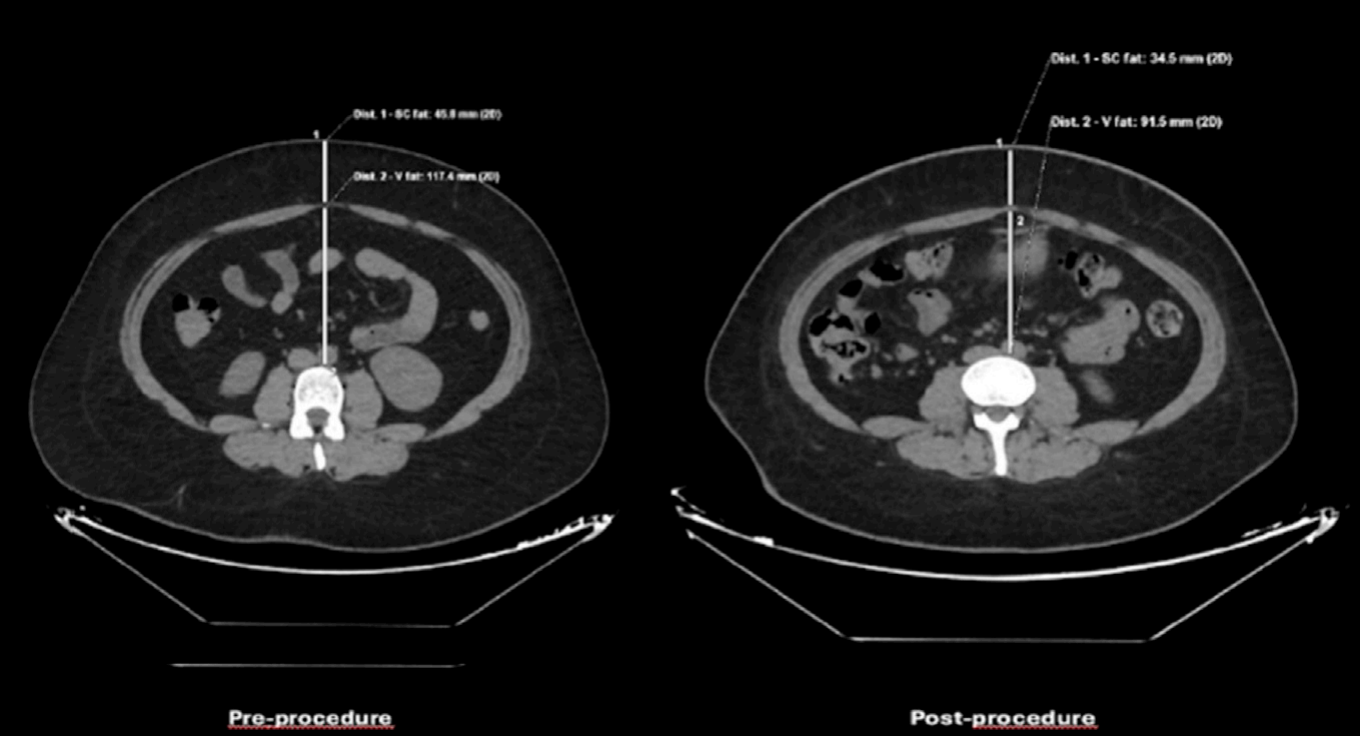
Pre-procedure and post-procedure measurements of visceral fat and subcutaneous fat tissue thickness on a patient’s CT scan.

Abdominal computed tomography (CT) scans within 1 month before and after the procedure were utilized when available. Fat tissue measurements were obtained from an axial CT slice at the level of the L3 vertebra. Visceral fat (V fat) was measured from the ventral aspect of the vertebra to the rectus sheath in the anterior-posterior direction, while subcutaneous fat (SC fat) was measured as the distance between the rectus sheath and skin level. All CT-based measurements were conducted by an expert radiologist (Figure 1).

### Ethical Considerations

Written informed consent was obtained from all patients for the endoscopic procedure. This study adhered to the principles outlined in the 1964 Helsinki Declaration and its subsequent amendments. Ethical approval was granted by the local ethics committee of Ankara Bilkent City Hospital (October 11, 2023, No. E2-23-4728).

### Follow-Up

Patients were scheduled for follow-up visits at 2 and 6 months post procedure. These follow-ups included detailed gastroenterological and dietetic evaluations performed by experts.

### Endoscopic Procedure

All endoscopic procedures were performed by 2 expert endoscopists in a dedicated endoscopy suite using 2 endoscope models (GIF 170; Olympus Optical Co., Tokyo, Japan, and EG 530WR; Fujinon, Tokyo, Japan). Patients underwent the procedure under anesthesia in the supine position. Sedation was achieved with midazolam and pethidine hydrochloride, with propofol administered as needed.

The endoscopic examination included a comprehensive evaluation of the upper gastrointestinal system. For patients without contraindications, a gastric balloon was inserted and inflated with 500 mL saline solution mixed with methylene blue within the gastric corpus. Balloon positioning was confirmed endoscopically, and the procedure was concluded. All patients were discharged on the same day.

Since gastric balloons are not covered by state insurance, patients are responsible for the cost in almost all cases. Consequently, the selection of the brand is entirely at the patient’s discretion.

### Statistical Analysis

The data were analyzed using IBM SPSS Statistics version 26 (IBM SPSS Corp.; Armonk, NY, USA). The normality of data distribution was assessed using the Kolmogorov–-Smirnov and Shapiro–Wilk tests. For comparing patient weights at different time points of the procedure, the Friedman test was applied for normally distributed data, followed by Bonferroni-corrected Dunn tests for multiple comparisons. Paired two-sample *t*-tests were used to analyze normally distributed variables. For variables that did not follow a normal distribution, the Wilcoxon signed-rank test was employed. The results were expressed as mean ± SD or median (minimum–maximum) for quantitative variables and as frequency (percentage) for categorical variables. Statistical significance was set at *P* < .05.

## Results

A total of 65 patients underwent endoscopic IGB therapy during the first season, encompassing 65 procedures, with procedure durations ranging between 8 and 15 minutes. One patient experienced severe nausea 2 days after IGB placement, and 2 patients were excluded from the study due to non-attendance at follow-up visits. As a result, the study was conducted with a total of 62 patients. The mean age of the patients was 37.89 years (range: 19-63), with 75.4% of the cohort being female. The mean body weight of the patients was 103.29 ± 16.2 kilograms (kg), and 27.4% of the patients had a BMI ≥ 40. The demographic and baseline characteristics of the patients are summarized in Table 1.

The BariGlobe 6-Month (Adjustable) gastric balloon was used in 56 patients (90.3%), the Endalis-End ball gastric balloon in 4 patients (6.5%), and the MedSil™ gastric balloon in 2 patients (3.2%). Gastric balloon placement was successfully performed in all patients initially included in the study (65/65, 100%). Only 1 patient (1.5%) required early removal of the IGB after 2 days due to severe nausea, and no other complications were observed during the 6-month follow-up period.

The body weight measurements of the patients during follow-up are presented in Table 2. The median body weight of the patients prior to the procedure was 104.5 kg, which decreased to 88 kg at the end of the 6-month follow-up period. After 6 months of IGB treatment, the calculated BMI values were significantly reduced (*P* < .001).

When examining the visceral fat (V fat) and subcutaneous fat (SC fat) tissue thickness of the patients before the procedure and 6 months after treatment (Table 3), a statistically significant reduction was observed in V fat tissue thickness following gastric balloon removal (*P* = .032). The mean V fat tissue thickness decreased from 109.97 ± 38.74 before the procedure to 101 ± 31.04 after balloon removal. While the mean SC fat tissue thickness of the patients before the procedure was 38.34 mm, the mean SC fat tissue thickness after the gastric balloon was removed was lower at 30.31 mm (*P* < .001).

## Discussion

This prospective single-center study evaluates the short-term outcomes of endoscopic IGB therapy in a cohort of obese patients. The results demonstrated significant weight loss and reductions in BMI over a 6-month follow-up period, reinforcing the efficacy of IGB therapy as a viable non-surgical intervention for weight management. The findings align with previous studies reporting substantial short-term weight reductions with IGB therapy. For instance, Saber et al^12^ conducted a meta-analysis of randomized controlled trials and found that patients with IGB therapy achieved a mean percentage total body weight loss of approximately 13.16% over 6 months. Similarly, a systematic review by Kotinda et al^14^ revealed an average weight reduction of 10-15 kg within 6 months of IGB placement. In another study by Genco et al^15^, patients achieved an average weight loss of 15.2 ± 8.7 kg after 6 months, with significant BMI reductions. In this study, the median body weight decreased from 104.5 kg pre-procedure to 88 kg at 6 months, corresponding to a mean BMI reduction from 37.59 to 32.79 kg/m^2^ (*P* < .001). These outcomes highlight the potential of IGB therapy as a valuable option for patients who may not be suitable candidates for bariatric surgery or who require preoperative weight reduction to improve surgical outcomes. Additionally, these results further validate the efficacy of IGB therapy as observed in diverse clinical settings and across varying patient populations. A recent study comparing IGB and endoscopic sleeve gastroplasty (ESG) demonstrated differences in weight loss outcomes and metabolic effects, offering valuable insights into the efficacy of these endoscopic bariatric procedures.^16^ While ESG appears to yield somewhat superior results, the accessibility and cost considerations of this technique suggest that IGB remains a viable and effective option for obesity treatment.

While this study demonstrates substantial short-term efficacy, the long-term sustainability of weight loss following IGB removal remains a critical question.^17^ Studies with extended follow-up have reported variable results regarding weight regain post-balloon removal, highlighting the need for integrated dietary and behavioral interventions to enhance long-term success.^13^^,^^18^

In addition to reductions in body weight and BMI, significant changes were observed in SC fat thickness, decreasing from a mean of 38.34 mm to 30.31 mm. Similarly, V fat thickness showed a modest but statistically significant reduction (*P* = .032). These findings are consistent with previous research reporting significant reductions in abdominal adipose tissue components, including both SC and V fat. For instance, markedly decreased levels of total abdominal adipose tissue, abdominal SC adipose tissue (AbSAT), and V adipose tissue (VAT) were noted at 6 and 12 months following bariatric surgery. Additionally, the total percentage reduction of VAT was significantly greater compared to the reduction in AbSAT, with the ratio of AbSAT/VAT increasing from 4.1 ± 1.7 preoperatively to 6.2 ± 3.1 at 12 months (*P* < .001).^19^ These findings suggest that IGB therapy, while targeting weight loss, may also favorably influence fat distribution, potentially contributing to improved metabolic health outcomes.

The safety profile of IGB therapy in this study was consistent with prior literature,^20^^,^^21^ with no major complications reported. One patient (1.5%) required balloon removal due to severe nausea, highlighting the importance of close monitoring and patient education regarding potential side effects. The absence of other significant adverse events reinforces the procedural safety of IGB therapy in appropriately selected patients. Despite its overall safety, the tolerability of IGB therapy can vary widely among patients.^22^ Commonly reported adverse effects, such as nausea, vomiting, and abdominal discomfort, may limit its acceptability.^22^^,^^23^ Advances in balloon design and improved patient management protocols could further enhance the tolerability and overall patient experience.

This study has several limitations that warrant consideration. First, the relatively short follow-up period of 6 months limits the ability to evaluate long-term weight maintenance and metabolic outcomes. Second, CT findings were not available for all patients, potentially limiting the generalizability of the observations regarding liver and fat tissue changes. Third, the patients’ 6-month biochemical values, such as cholesterol and HbA1c, could not be measured due to the lack of an appropriate indication. Consequently, the changes in these values before and after treatment could not be assessed. Additionally, this single-center design may not fully capture the variability in outcomes observed across diverse patient populations and clinical settings. Future multicenter studies with larger sample sizes and extended follow-up durations are necessary to validate and expand upon the findings.

Despite these limitations, this study has several notable strengths. The prospective design and standardized protocol for IGB therapy enhance the reliability of the findings. The inclusion of comprehensive assessments, including CT-based fat tissue analysis, provides valuable insights into the metabolic and structural changes associated with IGB therapy. Furthermore, the involvement of 2 experienced endoscopists ensures procedural consistency and reduces operator-dependent variability.

The findings of this study have important clinical implications for the management of obesity. Intra-gastric balloon therapy represents a minimally invasive, reversible option that can achieve significant short-term weight loss while avoiding the risks associated with bariatric surgery.^24^ As highlighted in this study,^25^ 25% of gastroenterologists in this country do not consider endoscopic techniques to be effective for obesity treatment. It is important to underscore that endoscopic IGB therapy, which is associated with a low risk of complications and offers significant weight loss, serves as a valuable alternative or bridging option in the management of obesity. To maximize the benefits of IGB therapy, a multidisciplinary approach involving dietitians, psychologists, and primary care providers is essential. Ongoing support and education can help patients maintain weight loss following balloon removal and address underlying behavioral and lifestyle factors contributing to obesity.

In conclusion, this study demonstrates the efficacy and safety of IGB therapy in achieving significant short-term weight loss and reductions in adipose tissue thickness. While the findings are promising, further research is needed to explore the long-term metabolic benefits, sustainability of weight loss, and optimal strategies for patient selection and management. Intra-gastric balloon therapy represents a valuable addition to the armamentarium of obesity treatments, offering a minimally invasive alternative for patients who are not candidates for surgical interventions.

## Figures and Tables

**Table 1.** Demographic and Baseline Characteristics of the Patients

	n = 62
**Age**	37.89 ± 10.68
**Sex**	
Female	46 (74.2)
Male	16 (25.8)
**Education Status**	
Elementary school	10 (16.1)
High school	20 (32.3)
Collegian	32 (51.6)
**Body Mass Index, ** *kg/m^2^*	
30-34	20 (32.3)
35-39	25 (40.3)
≥40	17 (27.4)
**Body Weight, ** *kilograms*	103.29 ± 16.2
**HOMA-IR**	3.97 ± 2.38
**HbA1c ** *%*	5.5 ± 0.76
**Total Cholesterol, ** *mg/dl*	180.95 ± 32.1
**LDL, ** *mg/dl*	107.85 ± 28.08

Mean ± SD, n (%).

HbA1c, glycated hemoglobin; HOMA-IR, homeostatic model assessment of insulin resistance; LDL, low density lipoprotein.

**Table 2.** Body Weight and Body Mass Index Measurements Before and After the Procedure

	Mean ± SD	Median (min.-max.)	*P*
Body weight, pre-procedure, kilograms	103.29 ± 16.2	104.5 (76-156)	**<.001^f^**
Body weight, after 2 weeks,kilograms	97.98 ± 15.49	99.5 (72-147)	
Body weight, after 2 months,kilograms	92.75 ± 14.78	94 (68-131)	
Body weight, after 6 months, kilograms	88.73 ± 15.27	88 (61-137)	
Pre-procedure BMI	37.59 ± 5.19	37.35 (30-55)	**<.001^w^**
BMI after 6 months	32.79 ± 4.95	32.3 (23.5-51.3)	

Median (minimum.-maximum).

BMI, body mass index.

^f^Friedman test.

^w^Wilcoxon test.

p< 0.05

**Table 3.** Comparison of V/SC Fat Tissue Thickness Values of Patients Before and After the Procedure

	Mean ± SD	Median (min-max)	Test Statistics	*P*
Pre-procedure SC fat tissue thickness, (n = 29)	38.34 ± 12.56	40 (0-67)	7.096	**<.001^t^**
SC fat tissue thickness after removal of balloon (n = 29)	30.31 ± 9.77	32 (0-48)		
Pre-procedure V fat tissue thickness, (n = 29)	109.97 ± 38.74	113 (0-177)	−2.138	**032^w^**
V fat tissue thickness after removal of balloon (n = 29)	101 ± 31.04	100 (0-170)		

SC, subcutaneous; V, visceral.

^t^paired two sample *t* test.

^w^Wilcoxon test.

## Data Availability

The data that support the findings of this study are available on request from the corresponding author.
